# Development and Evaluation of Fluconazole Co-Crystal for Improved Solubility and Mechanical Properties

**DOI:** 10.3390/pharmaceutics17030371

**Published:** 2025-03-14

**Authors:** Ritu Rathi, Inderbir Singh, Tanikan Sangnim, Kampanart Huanbutta

**Affiliations:** 1 Chitkara College of Pharmacy, Chitkara University, Rajpura 140401, India; ritu.rathi@chitkara.edu.in (R.R.); inderbir.singh@chitkara.edu.in (I.S.); 2 Faculty of Pharmaceutical Sciences, Burapha University, Mueang Chonburi 20131, Thailand; tanikan@go.buu.ac.th; 3 Department of Manufacturing Pharmacy, College of Pharmacy, Rangsit University, Mueang Pathum Thani 12000, Thailand

**Keywords:** co-crystal, antifungal, solubility, tabletability, compaction, compression

## Abstract

**Background**: Fluconazole (FLZ) is a broad-spectrum anti-fungal drug presenting poor flowability, mechanical properties, and limited aqueous solubility. These issues pose challenges for the handling and manufacturing of dosage forms of FLZ. The current research aimed to develop fluconazole co-crystal (CC) for improving its aqueous solubility, flowability, and mechanical properties. (2) **Methods:** The fluconazole benzoic acid (FLZ-BA) co-crystal was prepared using the solvent evaporation technique. The prepared co-crystal was characterized for drug content, solubility, anti-fungal activity, dissolution, and stability. DSC (Differential Scanning Calorimetry), PXRD (Powder X-Ray Diffraction), SEM (Scanning Electron Microscopy), and FTIR (Fourier Transmission Infrared) spectroscopy were carried out to confirm the co-crystal formation. The co-crystal was further evaluated for their flow characteristics and mechanical properties via CTC (compressibility, tabletability, and compactibility), Heckel, and Kawakita analysis. (3) **Results**: The CC showed 69.51% drug content and 13-fold greater aqueous solubility than pure FLZ. The DSC thermogram showed a sharp endothermic peak between the parent components, a distinct PXRD pattern was observed, and the SEM analysis revealed a different morphology, confirming the formation of co-crystal (new crystalline form). The CC showed immediate drug release and was found to more stable, and less hygroscopic than FLZ alone. The CC revealed better flowability, tabletability (tensile strength), compressibility, and compactibility. Moreover, Heckel and Kawakita analysis indicated the co-crystal to deform plastically, favoring improved compression. (4) **Conclusions**: The immediate drug release capabilities, improved hygroscopic stability, solubility, better antifungal activity, and flowability make FLZ-BA co-crystal a suitable candidate for the preparation of an immediate drug release dosage form. The study also revealed the application of co-crystal for improving the flowability and mechanical properties.

## 1. Introduction

A majority of active pharmaceutical ingredients (APIs) on the market exhibit poor physicochemical properties, particularly in terms of solubility, dissolution, and stability [1,2]. This presents significant challenges in achieving optimal therapeutic efficacy for these drugs. To address such issues, co-crystallization is one of the preferred strategies for enhancing the solubility and bioavailability of poorly soluble drugs through crystal engineering principles, without altering the chemical structure of the API [3,4]. Apart from these, co-crystal can improve the mechanical properties of API as well. Co-crystallization is a more recent approach aimed at enhancing the solubility and stability of drugs [5]. Co-crystals are formed by combining an API and a GRAS (Generally Recognized as Safe) coformer in a stoichiometric ratio, resulting in a crystalline lattice [6]. In general, a powder should have sufficient mechanical properties, which includes elasticity, plasticity, hardness, etc., so as to avoid tablet defects (such as fragmentation, cracking, and capping) [7]. Co-crystals are capable of improving the mechanical properties of API which are critical for tablet formulation. The mechanical properties of APIs are altered by changing their crystal structure rearrangement. This gives increased hardness, tabletability, and powder flow, thus making it easy to process and formulate into various dosage forms [8]. Additionally, co-crystal improves the stability of the API, thus reducing deterioration and increasing shelf life. For instance, when co-crystallized with saccharin, indomethacin, a poorly flowing powder, showed enhanced tabletability and decreased hygroscopicity. This improved mechanical property was due to a change in crystal packaging, leading to stronger intermolecular interactions [9,10]. Moreover, favipiravir is also known for its poor tabletability, which was enhanced by co-crystal formation with theophylline, saccharin, 5-fluorouracil, and piperazine. All the co-crystals showed improved tabletability except the favipiravir–theophylline cocrystal. The improved tabletability was due to altered crystal packaging that facilitated plastic deformation during compaction [11].

Fluconazole (FLZ) is a bis-triazole derivative-based broad-spectrum antifungal agent for managing systemic/superficial fungal infections and is available in various forms, including capsules, tablets, liquids, and intravenous solutions [12,13]. However, fluconazole has some limitations, such as limited aqueous solubility, poor flowability, and hygroscopicity, which make it difficult to formulate and manufacture. The poor flowability and mechanical properties of FLZ pose challenges for manufacturing tablet dosage forms by direct compression [14]. Several fluconazole co-crystals have been developed mainly to address the issue of limited solubility and dissolution. A study reported the co-crystal of FLZ with hydroxybenzoic acid and demonstrated enhanced dissolution rate [15]. Surov and colleague, depicted the solubility of CC at pH 5.7 [16] and another research prepared FLZ co-crystals with 2-chloro-5-nitrobenzoic acid and evaluated for computation analysis and interaction studies [17]. The CC of FLZ and dicarboxylic acids, aromatic carboxylic acid, ferulic acid, caffeic acid, and glycine was determined. A thorough literature survey revealed that the issue with poor stability, flowability, and mechanical properties of FLZ has not been explored precisely. Co-crystallization is a simple, economical, scalable, reproducible approach for improving the solubility [18,19,20], dissolution [21,22], stability [23], mechanical properties [24], flowability [25], and compressibility of pharmaceutical active ingredients. Former investigations were successful in obtaining co-crystals that can improve the mechanical properties of APIs, including paracetamol [26,27], carbamazepine [28,29], and telmisartan [30]. Bhatt et al. created co-crystals of metformin and sodium salicylate. Since metformin is a hygroscopic drug and exhibits poor tabletability, the co-crystal of metformin and sodium salicylate demonstrated improved tabletability via improved plastic deformation and intermediate elasticity and stiffness [31]. Co-crystal formation can reduce the hygroscopicity of fluconazole, making it less likely to absorb moisture from the air. This can improve the stability of fluconazole formulations and prevent them from becoming sticky or clumpy. Considering the potential of co-crystals, an attempt has been made to formulate FLZ co-crystals.

The current research aimed to improve the limited solubility, stability, and mechanical properties of fluconazole by crystal engineering. The co-crystal of FLZ-BA was prepared using the solvent evaporation technique. The obtained CC were characterized for drug content, solubility, dissolution, stability, hygroscopicity, and in vitro antifungal activity. The CC was also characterized using X-ray diffraction, DSC, and SEM analysis. The properties of co-crystal were compared with parent components. Moreover, the CC was evaluated for studying the bulk deformation behavior using compressibility (compaction pressure vs. porosity plot), tabletability (compaction pressure vs. tensile strength plot), and compactability (porosity vs. tensile strength) (CTC) analysis. In addition to this, the critical analysis of tableting and compressional characteristics was evaluated using the Heckel and Kawakita equation. The resulting co-crystal showed improved the mechanical properties of fluconazole.

## 2. Materials and Methods

### 2.1. Materials

Fluconazole was kindly supplied as a gift sample by Kwality Pharmaceuticals Ltd. Amritsar, India, and Benzoic acid was purchased from Sigma Aldrich, Burlington, Massachusetts, USA. All the excipients/reagents utilized in the study were of analytical grade.

### 2.2. Preparation of Co-Crystal

The co-crystals of fluconazole (FLZ) and benzoic acid (BA) were prepared by a solvent evaporation technique. The equimolar ratios of drug and coformer were solubilized in solvent (1:1 ratio of methanol and water). The resulting solution was sonicated (Probe Sonicator, Pro 656/900/1200, Chennai, Tamil Nadu, India) for 5 min, filtered, and transferred to a clean test tube, sealed with aluminum foil with 2–3 holes for the evaporation of solvent for a few days at room temperature (25 °C). The co-crystals that appeared were rapidly surface-dried and stored in a desiccator over silica pellets at room temperature for further studies [32].

### 2.3. Evaluation of Co-Crystal

#### 2.3.1. Solubility and Drug Content

Solubility studies were performed on pure drugs, the physical mixture (PM) of both the parent components, and co-crystal. The study was performed by suspending a sufficient amount of powder in 10 mL of distilled water and was kept in an orbital shaker (REMI, C-24 BL, Mumbai, Maharashtra, India) at 150 rpm, 37 ± 0.5 °C, for 24 h. After 24 h, the solution was filtered through Millipore filter paper (0.45 µm) and the amount of drug dissolved was analyzed using a UV spectrophotometer (Systronics, 2202, Ahmedabad, India) at 259 nm. The drug content was analyzed by dissolving 10 mg of co-crystal powder in a sufficient amount of methanol and the volume was made up to 100 mL with distilled water. The resulting solution was filtered and analyzed spectrophotometrically. The procedure was performed in triplicates [3].

#### 2.3.2. Differential Scanning Calorimetry

The thermal characteristics of the drug, coformer, physical mixture, and co-crystal were studied using DSC (PerkinElmer Thermal Analysis, DSC 4000, Waltham, MA, USA). Before starting the experiment, the instrument was calibrated for temperature and heat flow with high-purity indium. Then, 1–5 mg of the sample was placed in an aluminum crimping pan and heated in the presence of nitrogen gas at the rate of 5 °C/min from 0 to 300 °C.

#### 2.3.3. Thermogravimetric Analysis

TGA was performed on a Mettler-Toledo TGA/SDTA 851e instrument. Approximately 5–7 mg of the sample was heated from 25 to 400 °C at 10 °C/min under nitrogen purge.

#### 2.3.4. PXRD

The PXRD analysis was performed on Malvern Panalytical, PW 3040/60 (Almelo, The Netherlands) with Cu K∝ radiation (1.54 Å) at 40 kV and 7.5 mA with a step size of 0.02°. The samples were scanned between 4 and 60° (2θ). Before the initiation of the experiment, the instrument was calibrated using silicone standard. All the sample data were collected at ambient temperatures.

#### 2.3.5. SEM

The morphology of the powder samples was examined using a scanning electron microscope (Jeol-JSM 6510 LV, Tokyo, Japan). The powdered sample was placed over a specimen stub using double-sided adhesive carbon tape and coated with gold-palladium utilizing sputter coater SCD 005 (Edward, S-150, New York, NY, USA) for about 100 s at 30 mV. The images were captured over varied magnification ranges of 500× to 2000×.

#### 2.3.6. FTIR

The physiochemical interactions of CC were determined using FT-IR analysis. The FTIR spectrophotometer (Alpha Bruker, IFS 66/S, Ettlingen, Germany) was employed for FTIR studies. The dry potassium bromide was mixed with samples in the ratio 1:100 and compressed to form pellets. The samples were scanned within the range of 4000 cm^−1^ to 600 cm^−1^ wavenumber and 2 cm^−1^ resolution.

#### 2.3.7. In Vitro Anti-Fungal Activity

The antifungal test was performed using the cup-plate method against strain *Candida albicans*. The test organism suspension was prepared in a sterile normal saline solution. All the testing samples were prepared in 0.5% dimethyl sulfoxide at 100 mg/mL concentration and sterilized followed by the preparation of dilutions (50 mg/mL, 25 mg/mL, 12.5 mg/mL, and 6.25 mg/mL). The medium used was PDA (potato dextrose agar). A 6 mm die was impregnated and 20 μL of sample solution was mounted onto inoculated agar. The results obtained were in the form of the zone of inhibition [33,34].

### 2.4. Powder Evaluation

#### 2.4.1. Powder Flow Properties

Powder samples of FLZ, BA, PM, and CC, were subjected to measurement of different micrometric parameters, including Hausner’s ratio (HI), Carr’s index (CI), and angle of repose. The HI and CI were derived using Equations (1) and (2). The fixed funnel method was used to determine the repose angle, utilizing Equation (3) [35].

(1) $$Carr\text{’}s\;Index=\frac{Tapped\;density-Bulk\;density\;}{Tapped\;density}\times 100$$

(2) $$Hausner\text{’}s\;Ratio=\frac{Tapped\;density}{Bulk\;Density\;}\times 100$$

(3) $${tan}^{-1}\theta =\frac{h}{r}$$

#### 2.4.2. Compaction Studies

The compaction of the powder samples was studied by assessing its tabletability, compressibility, compactibility, and elastic recovery. The compaction studies were performed for all four solids FLZ, BA, PM, and CC. The tabletability was assessed by making powder compacts under different compaction pressures (25–150 MPa) using a Manual Hydraulic press (Specac^®^ Atlas, Z803103, Mainz, Germany). The compacts were stored for 24 h to relax residual stress after ejection. After 24 h, the breaking force, F (in MPa), was measured using a digital harness tester (Electrolab Hardness tester, EBT-2PRL, Mumbai, India), and tensile strength, T (in MPa), was calculated using Equation (4), where H is the thickness (mm) and the diameter is D (mm). F is the breaking Force of compacts. Tabletability was expressed using a tabletability plot between compaction pressure and tensile strength.

(4) $$Tensile\;strength=\sigma =\frac{2F}{\pi DH}$$

Compressibility was expressed by a compressibility plot between porosity and compaction pressure. The porosity (έ) of the compact was measured by utilizing compact density and powder true density, as shown in Equation (5) [29].

(5) $$Tablet\;Porosity=1-\left[\frac{\frac{tablet\;weight}{tablet\;volume}}{true\;density\;of\;powder}\;\right]\times 100$$

Compactibility was expressed by a compactibility plot between compact porosity and tensile strength [36].

The thickness, diameter, and hardness of compacts were measured as soon as it was ejected from the die hole. The tablets were allowed to rest for almost 24 h, and the diameter was measured again using a digital vernier caliper (Mitutoyo Digimatic Vernier Caliper, 500-196-20, Kanagawa, Japan). The axial elastic recovery % was measured by using an initial diameter (D*o*) and the diameter after storage (D) using the following Equation (6).

(6) $$Elastic\;recovery\;(ER\%)=\frac{D-D_{O}}{D}$$

The deformation mechanism of the powders was discovered by carrying out Heckel and Kawakita analysis. The Heckel equation is widely used for relating the relative density, D (ratio of bulk density of powder to the particle density), of a powder bed during compression to the applied pressure, P. Mathematically, the Heckel equation is written as follows:

(7) $$\ln\frac{1}{1-D}=KP+A$$

K and A are the slope and intercept, respectively, of the linear region of the heckle plot. The reciprocal of K is the yield pressure, *P_y_* of the material indicating the onset of plastic deformation. From the intercept A, the relative density, *D_A_* can be calculated using Equation (8) [30], as follows:

(8) $$D_{A}=1-e^{-A}$$

The relative density of the powder at the point when the applied pressure equals zero, *D_O_* is used to describe the initial rearrangement phase of densification as a result of die filling. Relative density, *D_B_*, describes the phase of rearrangement at low pressures and is the difference between *D_A_* and *D_O_*:

(9) $$D_{B\;}=D_{A}-D_{O}$$

The Kawakita equation is used to study powder compression using the degree of volume reduction (C) [36] and mathematically is represented as Equation (10):

(10) $$C=\frac{V_{O\;\;\;}-V_{p}\;}{V_{O\;\;\;}}=\frac{abP}{1+bP}$$

Equation (10) can be rearranged as follows:

(11) $$\;\frac{P}{C}=\frac{P}{a}+\frac{1}{ab}$$

where *V_O_* is the initial bulk volume of the powder and *V_p_* is the bulk volume after compression. Constant a is equal to the minimum porosity of the material before compression while constant b is related to the plasticity of the material. The reciprocal of b gives the pressure term PK, which is the pressure required to reduce the powder bed by 50% [37].

### 2.5. In Vitro Drug Release

The dissolution studies were performed for pure drug, physical mixture, and co-crystal using Dissolution Apparatus Type II (Lab India, DS8000, Pune, Maharashtra, India) employed at 50 rpm for 2 h. Then, 900 mL distilled water, at 37 °C ± 0.5 °C, was used as a dissolution medium. An equivalent weight of co-crystal containing 100 mg of drug was dispersed in 900 mL of dissolution media. Aliquots of 5 mL of all samples were collected at predetermined time intervals and replaced with fresh dissolution media each time to maintain the sink conditions. The collected samples were filtered and analyzed using a UV spectrophotometer at 259 nm [38].

### 2.6. Stability

The hygroscopic stability was determined by weighing approximately 25 mg of the sample and kept in a desiccator overnight with silica pellets. The samples were then subjected to different humidity conditions from 10 to 100%. The change in mass was calculated at each RH using a calibrated analytical balance. The stability studies were performed to evaluate the considerable changes that can occur during the storage of co-crystal. The CC were kept for accelerated stability conditions, i.e., 40 °C ± 2 °C/75% RH ± 5% RH for 6 months in a programmable environment test chamber (Remi SC-16 PLUS, Mumbai, India). The stability and integrity of samples were evaluated by FTIR and PXRD analysis [39].

## 3. Results and Discussion

### 3.1. Solubility and Drug Content

The solubility of the drug, PM, and co-crystal was evaluated in distilled water, summarized in Table 1 below. The results indicate that co-crystal showed maximum solubility. It was observed that the co-crystallization of FLZ significantly improved the solubility of FLZ by 13 folds. The rank order for solubility after 24 h was CC > PM > FLZ. The co-crystal was further evaluated for %yield and the practical yield was found to be 81.31%. The drug content obtained was 69.51 ± 0.38%, which is found to be good and consistent in co-crystal samples.

### 3.2. Differential Scanning Calorimetry

**Figure 1:**
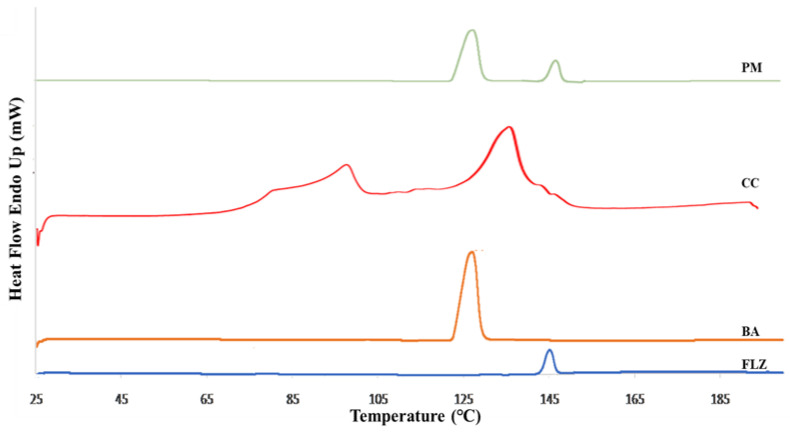
DSC thermogram of FLZ, BA, PM, and CC.

The DSC was employed for studying the thermal behavior of the samples and was performed for pure drug, coformer, physical mixture, and co-crystal, Figure 1. The DSC thermogram of FLZ and benzoic acid showed a single endothermic peak at 145 °C and 129 °C, depicting the melting point of FLZ and BA, respectively. The physical mixtures of the drug and coformer were prepared that exhibited two distinct endothermic peaks representing the characteristic melting points of plane compounds, thereby indicating the absence of co-crystal formation and compatibility between the drug and coformer. The DSC thermogram of FLZ-BA co-crystal displayed an endothermic dip at 136 °C, indicating the formation of a new crystalline form. Hence, it may be deduced that the supramolecular arrangement in the FLZ-BA co-crystal altered the melting point of the material. Furthermore, the endothermic peak at 102 °C could be ascribed to the released water molecule from the crystal lattice. Similar results were observed by Patel et al., where the melting point of diacerein and β-resorcylic acid co-crystal showed intermediate melting temperatures than parent components, and suggested that during the screening of the co-crystal, if the melting point of the system is intermediate, then there is a high possibility of co-crystal formation [40].

### 3.3. TGA

**Figure 2:**
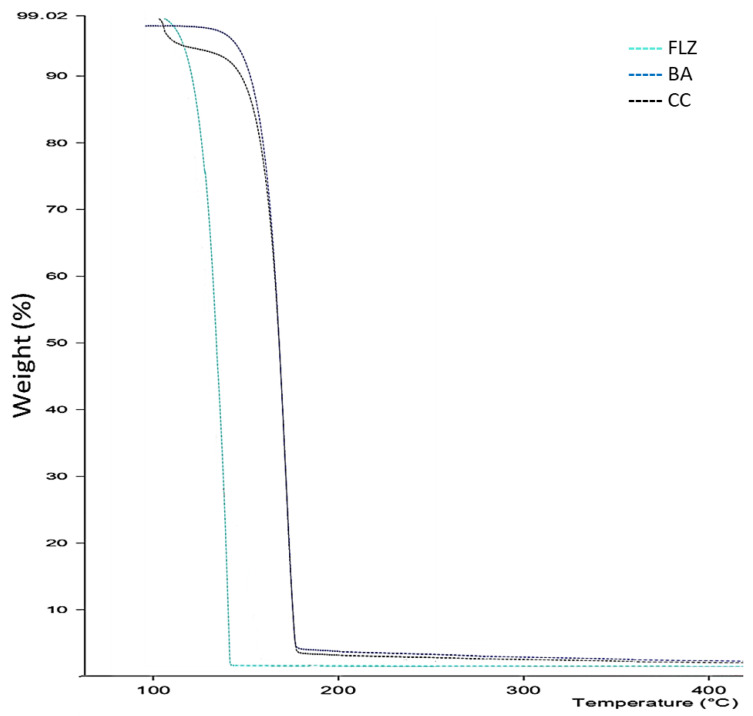
TGA profile of thermogram of FLZ, BA, and CC.

The TGA profile of FLZ-BA co-crystal and the native components is shown in Figure 2. The thermogram of FLZ and BA showed a single step decomposition. In contrast, the co-crystal indicated a two-step decomposition process with a total mass loss of 4.18% in the temperature range of 110–120 °C, which could be attributed to dehydration coinciding with a DSC thermogram endothermic peak at 102 °C. Moreover, the co-crystal exhibited excellent thermal stability (degradation temperature 225 °C) than parent FLZ, which undergoes decomposition over 160 °C, indicating the formation of a new crystalline solid form.

### 3.4. PXRD

**Figure 3:**
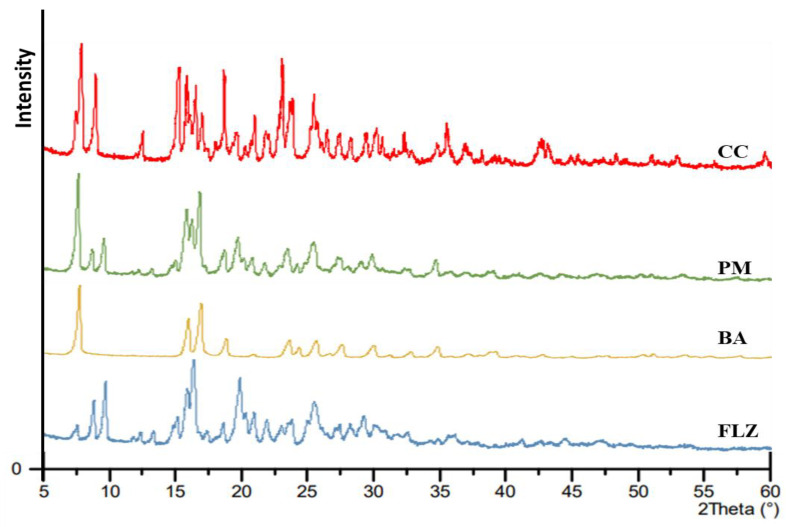
PXRD pattern of FLZ, BA, PM, and CC.

The formation of co-crystal was further confirmed by PXRD analysis. Figure 3 illustrates the PXRD patterns of FLZ, BA, PM, and CC. The diffractogram of pure FLZ showed characteristic peaks at 2θ values of 9.1°, 10.03°, 16.5°, and 20° whereas BA characteristic peaks were observed at 2θ angle 8.2°, 16.2°, 17.1°, and 19.8°. The physical mixture diffraction pattern revealed characteristic peaks of parent components (2θ = 8.07°, 10.05°, 16.2°, and 17.1°). The PXRD pattern of FLZ-BA co-crystal displayed a characteristic reflection at (15.3°, 15.9°,18.7°, 23.1°, and 25.5°), indicating newness in its crystalline phase, notably distinct from its precursor. Alterations in the number and intensity of crystalline peaks in the PXRD pattern of FLZ-BA co-crystal could be attributed to the interactions between the drug and coformer, indicating the formation of the co-crystal.

### 3.5. SEM

**Figure 4:**
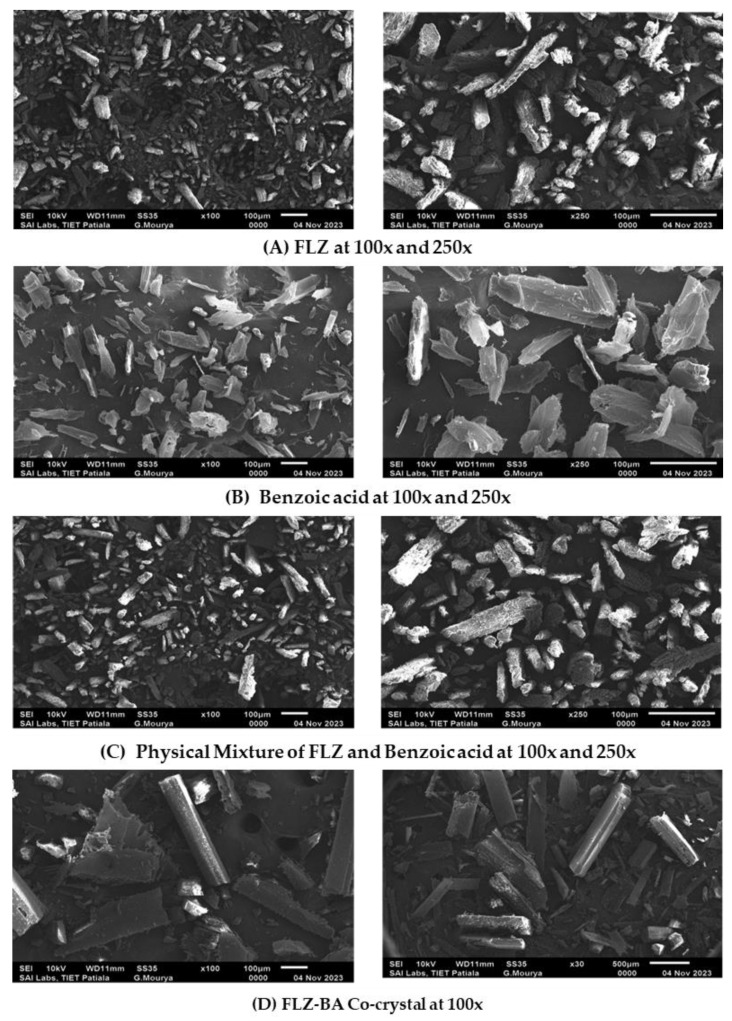
SEM images of (**A**) FLZ, (**B**) BA, (**C**) PM, and (**D**) CC.

The change in morphology of the drug was analyzed using SEM. The surface morphology of the drug, coformer, physical mixture, and co-crystal monographs are represented in Figure 4. Pure drug/FLZ showed grain-like crystalline particle agglomerates whereas benzoic acid appeared to be prism/platelet-like crystalline structures [41] and the co-crystal showed unique flat rod-shaped crystalline sticks [42]. To confirm the uniqueness of co-crystal structures, the morphology of the physical blends of the drug and coformer was also studied, which revealed the existence of parent components individually with no unique structure. The co-crystal showed a unique morphology, which was distinct from both the parent components. In summary, the DSC and PXRD results confirmed the formation of a new crystalline phase different from starting moieties and exhibited rod-like structures, as confirmed by SEM analysis.

### 3.6. FTIR

**Figure 5:**
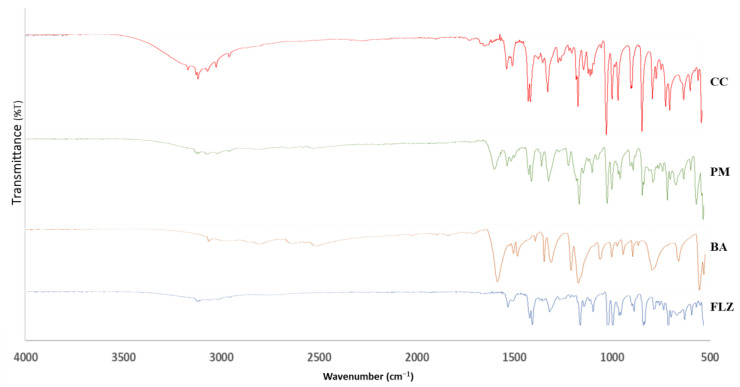
FTIR spectra of FLZ, BA, PM, and CC.

The FTIR spectra of FLZ, BA, PM, and CC are illustrated in Figure 5. The FTIR analysis was performed for identifying non-covalent interactions within the co-crystal. The FTIR spectra of FLZ depicted peaks at 3116 cm^−1^ (-OH stretching), 1618 cm^−1^ and 1513 cm^−1^ (aromatic ring C=C stretching), and 1501 cm^−1^ and 1418 cm^−1^ (triazole ring stretching) [43]. Meanwhile, benzoic acid represented bands at 3070 cm^−1^, representing -OH stretching and carbonyl stretch at 1729 cm^−1^. Co-crystal formation is generally indicated by studying the involvement of C=O and -OH functional groups, depicting characteristic peaks in the range of 1740–1600 cm^−1^ and 3600–3300 cm^−1^. The appearance of a characteristic peak at 1730 cm^−1^ indicates the participation of a carbonyl group in hydrogen bonding interactions, leading to the formation of a co-crystal. Additionally, there is an appearance of broad peaks in the range of 3300–2800 cm^−1^. This possibly indicates the formation of FLZ-BA co-crystal hydrates, wherein water molecules have occupied the hydrogen bonding sites of FLZ, significantly reducing the probability of water molecules approaching FLZ. This could be attributed to the hygroscopic stability of FLZ-BA co-crystal in comparison to pure FLZ. In agreement with PXRD, DSC, and SEM analysis, the FTIR analysis also suggested the formation of a new crystalline form.

### 3.7. In Vitro Anti-Fungal Activity

The cup-plate method determined the in vitro antifungal activity of FLZ, BA, PM, and CC against *Candida albicans*. The results indicated that both plane FLZ, PM, and cocrystals showed reduced growth for the organism, but the co-crystal signifies better inhibitory action than FLZ and BA alone. Table 2 shows the antifungal activity of FLZ, BA, PM, and CC. The highest zone of inhibition against *Candida albicans* was observed at 100 μg/mL (11 ± 0.1, 2 ± 0.2, and 20 ± 0.1 mm) for FLZ, BA, and CC. Also, the minimum zone of inhibition for CC was found to be 12.5 μg/mL, but for FLZ, it was observed to be 25 μg/mL.

### 3.8. Powder Evaluation

#### 3.8.1. Flow Characterization

The bulk density, tapped density, Carr’s index, Hausner’s ratio, and angle of repose were determined for fluconazole, benzoic acid, physical mixture, and co-crystal. The results are shown below in the Table 3.

The Hausner’s ratio and Carr’s index of CC were found to be 2.0 ± 0.09 and 1.08 ± 0.05, respectively, representing good flow characteristics. Meanwhile, pure drug and physical mixture showed poor to passable flow characteristics. In contrast, the co-crystal former also showed good flow characteristics. Similar results were demonstrated by Alatas et al., where FLZ-tartaric co-crystal showed improved flow characteristics [44].

#### 3.8.2. Compaction Studies

The true densities of pure drug and CC were 1.47 and 1.569 g/cm^3^, respectively, indicating that CC is relatively denser than pure drug. Compared to pure drug alone and physical mixture, the variation in co-crystal density indicates the increased compression behavior of CC [40].

**Figure 6:**
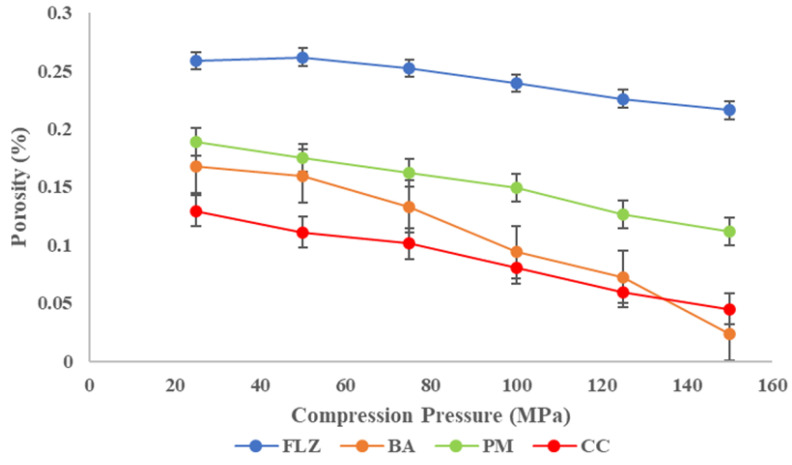
Compressibility plot of FLZ, BA, PM, and CC.

The compaction properties of all four compounds were studied by CTC profiling (compressibility, tabletability, and compactibility). Compressibility refers to the ability of powder to undergo a volume reduction under the influence of compaction pressure and is represented by the plot against the porosity of powder and compaction pressure [25,45]. The compressibility plot of all four compounds is shown in Figure 6. It is important to study the compressibility as it is closely related to the mechanical properties of tablets such as elasticity, hardness, tabletability, and compactibility. The higher compressibility of CC over parent compounds was observed at the entire compaction range. The compressibility followed the order CC > BA > PM > FLZ. The co-crystal showed the maximum compressibility, even at a low compaction pressure among all compaction.

**Figure 7:**
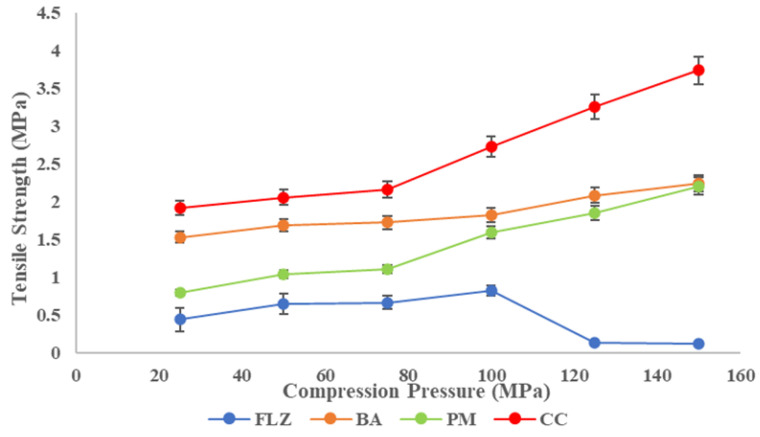
Tabletability plot of FLZ, BA, PM, and CC.

Tabletability is the ability of a powder to be compressed into a tablet of sufficient tensile strength under the impact of compaction pressure and is expressed by the tabletability plot between tensile strength (TS) and compaction pressure, P [46]. The tabletability plot of FLZ, benzoic acid, physical mixture, and CC is shown in Figure 7. The TS of all the compounds increased with increasing compaction pressure (25–150 MPa) and the tabletability order was CC > BA > PM > FLZ. Pure FLZ showed TS 0.7 MPa at 25 MPa pressure to 0.8 MPa at 100 MPa pressure, and beyond this pressure, fragmentation and capping occurred. Therefore, the tabletability of FLZ is poor. The crystal former showed sufficient tensile strength at higher pressures > 100 MPA pressures. The physical mixture showed improved tensile strength at increasing compaction pressures but the TS was 1.5 MPa at 100 MPa pressure; above this pressure, somewhat sufficient tensile strength was obtained, but capping and lamination were observed. In contrast, CC showed much-improved tabletability at the entire compaction pressure range. A minimum TS of 2 MPa has been proposed to maintain the integrity of pharmaceutical tablets. CC meets this criterion at a pressure of 50 MPa only. For CC, the tensile strength of >2 MPa was obtained with no capping and lamination; therefore, a formulation containing CC is not expected to have a problem with tableting.

**Figure 8:**
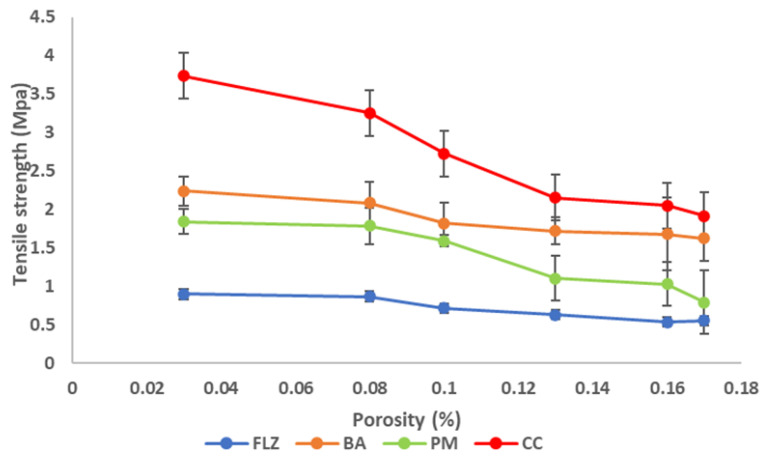
Compactibility plot of FLZ, BA, PM, and CC.

Compactibility is the powder’s ability to be compressed into tablets with sufficient tensile strength under the effect of densification and is expressed by the compactibility plot between tensile strength and porosity. The plot signifies the material’s bonding strength of the material [37]. The compactibility plots of FLZ, BA, PM, and CC are shown in Figure 8. According to various reported research porosity and tensile strength have an inverse relationship, and the same relationship was observed in the present work. As the porosity of the compounds decreases, the tensile strength increases linearly, depicting larger hardness and resistance to deformation. CC indicated a higher bonding area than pure drug and physical mixture, as CC exhibited low porosity at the entire compaction range that corresponds to increased tensile strength and mechanical properties of the compact.

**Figure 9:**
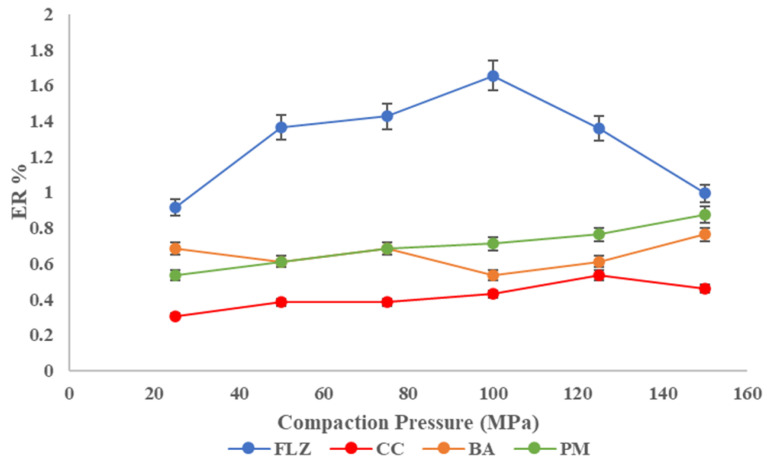
Elastic recovery plot of FLZ, BA, PM, and CC.

The percentage of elastic recovery (ER) could be used to estimate the elasticity of different pharmaceutical materials that will be compacted into tablets. Testing the elastic recovery percentage of active pharmaceutical ingredients or excipients can help reduce the possibility of capping or other problems during tablet formulation and manufacturing. The pure FLZ has a higher proportion of elastic recovery than the co-crystal, implying that the co-crystal has better tablet ability. The ER% is in the following order: FLZ > PM > BA > CC, as shown in Figure 9.

- Heckel analysis

**Figure 10:**
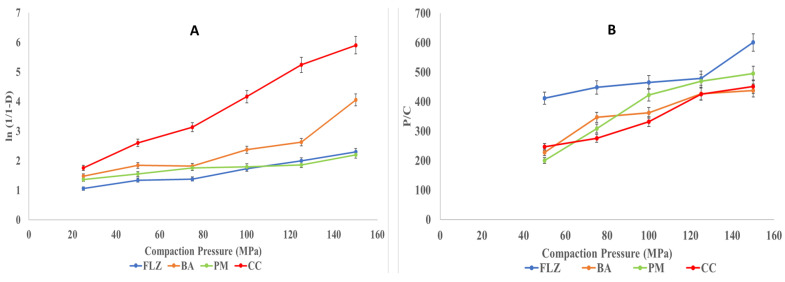
(**A**) Heckel and (**B**) Kawakita plots for FLZ, BA, PM, and CC.

The current study presents the densification of FLZ, BA, PM, and CC under the function of compaction pressure. The Heckel plot for the compounds is shown below in Figure 10A. In the Heckel plot, the correlation coefficient of R^2^ > 0.99 was observed in the case of CC, while FLZ, BA, and PM showed an R^2^ equal to 0.97, 0.83, and 0.93, respectively.

All the parameters derived from Heckel plots are tabulated in Table 4. The larger values of A and K are generally favorable for powder compaction, which indicates better compression behavior [40]. Among all the four solids, CC showed the maximum values for A and K, representing better plastic deformation and particle rearrangement. In contrast, yield pressure represents the deformation nature of crystal habit and is inversely proportional to the nature of crystals to deform plastically [37]. The lower value of *P_y_* among all four compounds indicates its plastic deformation and hence, better compressibility. The *D_A_* and *D_B_* representing initial particle rearrangement is higher for CC, which showed better tabletability of CC than other compounds.

- Kawakita Analysis

The Kawakita equation describes the relationship between the compression pressure (P) and degree of volume reduction (C). The plot of P/C is shown in Figure 10B for FLZ, BA, PM, and CC, which resulted in a linear relationship. The measurement of the initial relative density of the formulation when small pressure is applied to the powder bed is the *D_i_* value. The *D_i_* values were observed in the order CC > BA > PM > FLZ (Table 5). The higher *D_i_* values indicate denser compacts with better mechanical properties. The *P_k_* values are inverse measurements of total or overall plastic deformation [47] and the observed order was CC > PM > BA > FLZ. The lowest *P_k_* value was observed for CC, hence depicting the highest total plastic deformation.

### 3.9. In Vitro Drug Release

**Figure 11:**
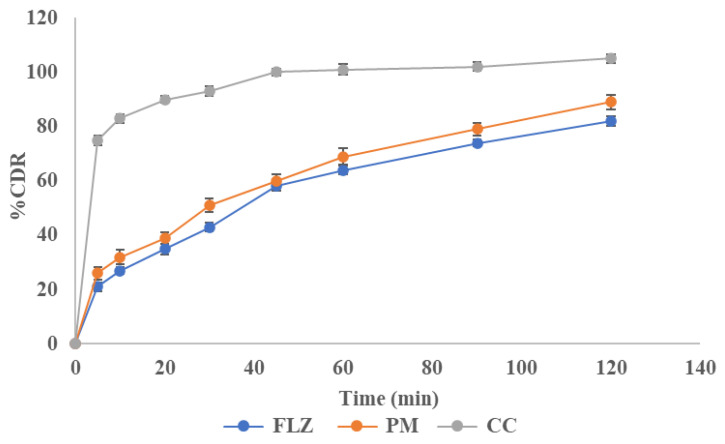
Dissolution profile of FLZ, PM, and CC.

A dissolution study was conducted to analyze the release behavior of FLZ from the pure drug, physical mixture, and co-crystal. About 75 ± 0.8% of the drug was released from co-crystals within 5 min and it was just 21.1 ± 0.57% and 31.9 ± 0.37% for the drug and physical mixture, respectively. The result showed an increased initial powder dissolution rate from the co-crystal and a higher drug release than pure drug and physical mixture. This increased release rate in the initial phases of dissolution will be beneficial in the formation of an immediate-release dosage form. This revealed that the FLZ-BA co-crystal system enhanced dissolution. The dissolution profile is shown in Figure 11.

### 3.10. Stability

**Figure 12:**
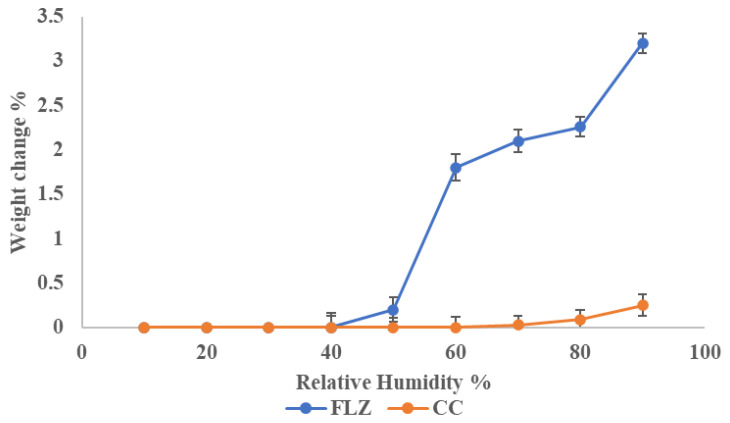
Percentage mass change in FLZ and CC at different RH.

From the literature survey, it was observed that FLZ converts to fluconazole monohydrate when exposed to RH > 40% due to the binding of water molecules at the hydroxy, N_3_, and N_6_ [15]_._ Hence, the current work was to focus on enhancing the stability of FLZ. For comparison, pure drug and co-crystal were exposed to different relative humidity conditions for one week and the change in mass was observed. The plot depicted a change in mass (%) with different humidities, as shown in Figure 12. The total mass change in FLZ was found to be 3.2% ± 0.4%, which can be classified as hygroscopic material, in accordance with European Pharmacopoeia. It was found that co-crystal was stable at accelerated humidity conditions and could be classified as slightly hygroscopic to above RH 80%. The increased stability of FLZ is because the crystal coformer occupied all the H-bonding sites in FLZ, reducing the portability of water to bind.

**Figure 13:**
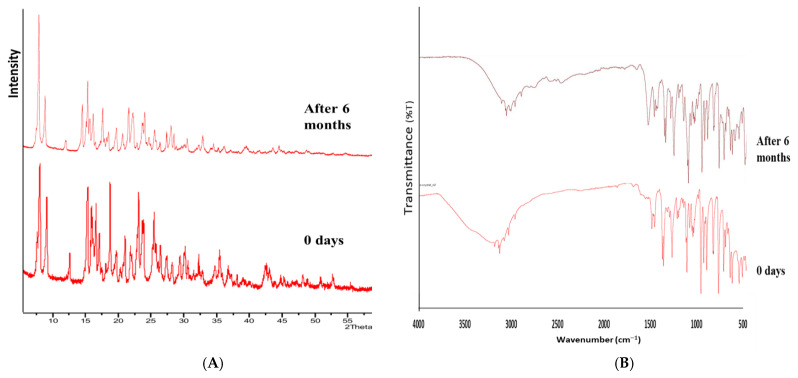
(**A**) PXRD and (**B**) FT-IR spectra of CC before and after 6 months.

After 6 months of accelerated stability conditions, no change in the morphology (color and texture) of co-crystal was observed. The drug content, solubility, and dissolution profiles of co-crystal before and after the study were almost similar. A similar trend was observed in FT-IR and PXRD data, before and after 6 months (Figure 13). This indicated that the co-crystal samples were stable.

## 4. Conclusions

The current research is focused on preparing pharmaceutical FLZ-BA co-crystals using a solvent evaporation technique following characterization by SEM, FTIR, DSC, and PXRD techniques. The co-crystal revealed improved aqueous solubility by 13 folds than FLZ alone. Furthermore, the pure FLZ and co-crystal were subjected to powder flow characterization and the co-crystal showed improved flow characteristics than FLZ alone. The FLZ-BA co-crystal compacts showed tensile strength >2 MPa, even at a compaction pressure of 50 MPa. The percentage elasticity of co-crystal was much lower than FLZ; hence, reducing the fragmentation and capping issues improved mechanical properties and better plastic deformation than pure FLZ. In addition, the Heckel and Kawakita analysis of co-crystal also points towards better plastic deformation and particle rearrangement. The improved tensile strength, reduced elasticity, and better plastic deformation of co-crystal point towards the formation of a co-crystal loaded-tablet without tablet defects.

Moreover, the in vitro antifungal tests showed the antifungal activity of co-crystal against *Candida albicans* improved by pure FLZ by 1.81 times. An increased powder dissolution was observed, representing almost more than 80 percent drug release in the initial phases of dissolution, suggesting the formation of immediate release formulations. Furthermore, the co-crystals were successful in reducing the hygroscopicity, and hence improved the stability of FLZ even at accelerated stability conditions. The current study suggested the co-crystallization technique’s potential to improve not just physicochemical properties but physicomechanical properties of pharmaceutical APIs. The approach can be applied to industries for addressing drugs with poor compressibility to convert them into directly compressible solids. The outcome of the current research will be more pronounced towards commercialization by exploring the structural characterization of the solids, in vivo characterization, and formation of suitable dosage forms in the futuristic plan.

## Figures and Tables

**Table 1:** Physicochemical characteristics of FLZ, PM, and CC.

S. No.	Compound	Drug Content (%)	Solubility (mg/mL)
1	FLZ	-	0.21 ± 0.03
2	PM	70.34 ± 0.86	0.28 ± 0.07
3	CC	69.51 ± 0.53	2.82 ± 0.08

**Table 2:** Antimicrobial efficacy as zone of inhibition (in mm) at different concentrations for FLZ, BA, PM, and CC. Samples against *Candida albicans*.

S. No.	Sample Code	Antimicrobial Efficacy as Zone of Inhibition (in mm) at Different Concentrations of Samples Against *Candida albicans*				
		100 (μg/mL)	50 (μg/mL)	25 (μg/mL)	12.5 (μg/mL)	6.25 (μg/mL)
1	FLZ	11 ± 0.2	9 ± 0.4	7 ± 0.2	0	0
2	BA	2 ± 0.6	0	0	0	0
3	PM	15 ± 0.4	8 ± 0.5	5 ± 0.8	4 ± 0.4	0
4	CC	20 ± 0.5	12 ± 0.2	10 ± 0.2	6 ± 0.6	0

**Table 3:** Flow characterization parameters for FLZ, BA, PM, and CC.

Parameters	FLZ	BA	PM	CC
Bulk density (g/cm^3^)	0.41 ± 0.02	0.41 ± 0.05	0.62 ± 0.02	0.68 ± 0.04
Tapped density (g/cm^3^)	0.83 ± 0.02	0.83 ± 0.04	0.83 ± 0.06	0.83 ± 0.07
True density (g/cm^3^)	1.47 ± 0.07	1.32 ± 0.08	1.45 ± 0.03	1.56 ± 0.05
Carr’s index (%)	50.0 ± 1.2	7.69 ± 1.6	21.87 ± 1.3	17.24 ± 1.5
Hausner’s ratio	2.0 ± 0.09	1.08 ± 0.05	1.28 ± 0.04	1.14 ± 0.07
Angle of repose (deg)	76.1 ± 1.2	31.28 ± 1.8	41.5 ± 1.4	33.0 ± 1.6
Type of Flow	very poor	good	passable	good

**Table 4:** Derived parameters for FLZ, BA, PM, and CC from Heckel plot.

	K	*P_y_* (MPa)	A	e^−A^	*D_A_*	*D_B_*
FLZ	0.0131	76.36	0.418	0.62	0.34	0.19
BA	0.021	46.96	0.82	0.65	0.37	0.15
PM	0.0112	89.29	0.66	0.51	0.35	0.16
CC	0.037	26.66	0.44	0.64	0.48	0.20

K is the slope of the linear regression plot; *P_y_* is the yield pressure; A is the intercept of the linear regression plot; *D_A_* is the relative density; relative density at porosity equals zero, and *D_B_* is the phase of rearrangement of low pressure.

**Table 5:** Derived parameters from Kawakita Plot for FLZ, BA, PM, and CC.

	*D_i_*	*P_k_*
FLZ	0.51	0.040
BA	0.76	0.009
PM	0.68	0.0214
CC	0.98	0.003

## Data Availability

Dataset available on request from the authors.
